# Identification of *OCT* Family Genes in Tomato (*Solanum lycopersicum*) and Function of *SlOCT20* Under Cold Stress

**DOI:** 10.3390/biology15020176

**Published:** 2026-01-18

**Authors:** Rui Lv, Fulei Mo, Yuxin Liu, Huixin Zhang, Mingfang Feng, Peiwen Wang, Mozhen Cheng, Shusen Liu, Zhao Liu, Xiuling Chen, Aoxue Wang

**Affiliations:** 1 College of Horticulture and Landscape Architecture, Northeast Agricultural University, Harbin 150030, China; lvrui34324080@163.com (R.L.); yuxinliu1223@163.com (Y.L.); wangpeiwen@neau.edu.cn (P.W.); neaucmz@163.com (M.C.); 2 College of Life Sciences, Northeast Agricultural University, Harbin 150030, China; neaumfl@163.com (F.M.); zhanghuixin_123@163.com (H.Z.); fengmingfang@neau.edu.cn (M.F.); 3 School of Life Science and Technology, Harbin Institute of Technology, Harbin 150001, China; 4 Shandong Shouguang Sanmu Seedling Co., Weifang 250013, China; shusenliu2023@163.com (S.L.); sanmuliuzhao@163.com (Z.L.); 5 Key Laboratory of Biology and Genetic Improvement of Horticultural Crops (Northeast Region), Ministry of Agriculture and Rural Affairs, Harbin 150030, China

**Keywords:** tomato, cold stress, *OCT*, cadaverine, VIGS

## Abstract

Plant organic cation transporters (OCTs) are involved in a variety of beneficial biological processes, such as cadaverine transfer in plants and soil, and play an active role in the formation of plant stress resistance. In this study, 52 *OCT* family genes were identified in tomato, and comprehensive bioinformatics analyses of these numbers, such as promoter *cis*-acting elements, gene mapping and collinearity, protein characterization and phylogenetic analysis. By analyzing the expression of tomato *OCT* family genes under cold and salt stresses using transcriptome data and qRT-PCR experiments, a key gene regulating cold stress tolerance, *SlOCT20*, was identified. Subcellular localization experiments indicated that SlOCT20 was mainly localized in the cell membrane. When the *SlOCT20* gene was silenced in tomato, the tolerance to cold stress was significantly reduced and oxidative stress was aggravated, indicating that this gene positively regulates the tolerance to cold stress in tomato.

## Simple Summary

Tomato, as a vegetable crop that prefers warmth, has relatively high requirements for planting temperature. Cold stress often occurs in tomato cultivation, especially in early spring and late autumn. Cold stress not only affects the growth, development and yield of tomatoes, but in severe cases, it can also lead to the direct death of tomato plants. Screening cold-resistant genes and analyzing their regulatory mechanisms of cold resistance are of great significance for enhancing the pertinence of stress-resistant breeding. This study identified the cold-resistant gene *SlOCT20* in cultivated tomatoes and analyzed the molecular mechanisms by which it regulates tomato cold resistance, providing a new regulatory mechanism of plant cold resistance.

## 1. Introduction

In complex environments, plants have evolved multiple mechanisms to adapt to stress. The transmembrane transport of cations plays a significant role in this process, including mineral absorption, osmotic pressure regulation, cell expansion, environmental and cellular signal transduction and stress tolerance [1]. OCTs are involved in a variety of biological processes. OCT proteins are not only present in animals but also widely found in higher plants.

OCT family proteins belong to the Solute Carrier 22A (SLC22A) superfamily and are involved in the transmembrane transport of organic cations [2]. The first reported *OCT* gene in plants was identified in *Phasolus vulgaris*, which is induced by drought and other stresses, but its substrate remains unknown [3]. In *Arabidopsis thaliana*, *BOU* is proposed to encode a mitochondrial acetylcarnitine transporter, and OCT1 has been shown to repress *BOU* transcription. The Arabidopsis *oct1* mutant showed an altered root sensitivity to carnitine, with higher lateral root density in the *oct1* mutant than in the wild type. Yeast mutant transformation experiments revealed that AtOCT1 may function as a carnitine transporter [4]. AtOCT1 protein is localized to the cell membrane, and is expressed in vascular tissues and lateral root initiation sites. *AtOCT1* promoter activity has also been detected in the vascular tissues of roots, leaves and flowers, suggesting that *AtOCT1* may play a role in transport in plants [5].

Soil contains organic cations such as cadaverine cation and carnitine zwitterion, and plants can synthesize carnitine and cadaverine under stress conditions [6,7]. The above organic cations in plants play an active role in plant development and stress adaptation [8]. Studies on the absorption and transport mechanism of beneficial organic cations in plants can deepen the theory of plant stress resistance. In this study, 52 *OCT* family genes were identified using the tomato genome version 4.0. The intron and exon distribution of all *OCT* family genes in tomato was visualized. The conserved structures and features of all OCT proteins were characterized. By constructing the OCT family protein phylogenetic tree and analyzing the collinearity relationship among *OCT* family genes, the evolutionary laws of *OCT* family genes in plants can be understood. In addition, the *cis*-acting elements of tomato *OCT* family genes in their promoters were statistically analyzed, and the expression patterns of tomato *OCT* family genes under salt and cold stress were clarified by transcriptome data analysis and qRT-PCR experiments. The function of *SlOCT20* in cold stress was resolved by silencing the *SlOCT20* gene. This study elucidated the evolutionary and functional differentiation of *OCT* family genes in tomato and clarified the association between *OCT* family genes and cold stress tolerance in tomato.

## 2. Materials and Methods

### 2.1. Planting Environment and Different Stress Treatments

The tomato variety used in this study is ‘Ailsa Craig’, and the tobacco (*Nicotiana benthamiana*) seeds were stored and provided by our laboratory. Seeds were sown in fertile soil and grown in the plant incubator. The soil composition consisted of 80% mass of uncontaminated field soil (obtained from Xiangyang Farm, Northeast Agricultural University, Harbin, China) and 20% mass of perlite, which was mixed and sterilized at high temperature for use. The sterilized soil was loaded into a 200 mL capacity plastic flowerpot, and the seeds were soaked in distilled water for 12 h before sowing. Plant incubator parameters: (illumination time: 15.5 h, illumination intensity: 25,000 lux, parameter of temperature: 25.5 °C; time of night: 8.5 h, parameter of temperature: 19.5 °C). When tomatoes reached 4 weeks of age, they were subjected to salt and cold stress treatments. The concentration of NaCl in the salt stress treatment was 100 mmol/L, the treatment was carried out by root irrigation (single irrigation, 200 mL per tomato plant) and the control group was treated with the same volume of distilled water. Cold stress treatment parameters: (illumination time: 15.5 h, illumination intensity: 25,000 lux, parameter of temperature: 4.0 °C; time of night: 8.5 h, parameter of temperature: 4.0 °C). The temperature of the control group was 25.5 °C during the day and 19.5 °C during the night, and other parameters were the same as those of the cold treatment group. The cooling mode of the cold stress treatment was direct cooling, that is, the plants were directly transferred to 4 °C. The control group of the VIGS experiment was tomato transfected with pTRV2-00 empty vector, and the stress treatment was the same as above. Tomato tissue samples were removed and snap frozen in liquid nitrogen for three biological replicates at 0 h, 0.5 h, 2 h, 4 h, 6 h, 12 h, 24 h and 48 h of stress, and these samples were used for subsequent experiments.

### 2.2. Identification of OCT Family Members in Tomato and Characters of OCT Proteins

Known OCT protein sequences in Arabidopsis were obtained from the TAIR database (https://www.arabidopsis.org/, accessed on 17 January 2025). The tomato genome file (version 4.0; from cultivated tomato Heinz 1706) and its annotation file were obtained from the Phytozome database (http://phytozome.jgi.doe.gov/pz/portal.html, accessed on 19 January 2025). All tomato DNA sequences were translated, and the resulting protein file was used as a library, in which BLAST proteins similar to the Arabidopsis OCT protein were identified using the BLASTp plugin in TBtools (v2.138), E value = 1 × 10^−5^. The HMM profile (PF00083) for the OCT protein family, retrieved from the Pfam database (accessed on 26 January 2025), was employed to screen the tomato protein sequences using TBtools software (v2.138) [9]. The threshold parameter is the default of the software. The intersection of the search results of BLAST and HMM was taken as the *OCT* family members in tomato. Tomato OCT family protein sequences were extracted and submitted to Expasy (https://web.expasy.org/, accessed on 5 February 2025) to analyze their characteristics.

### 2.3. Localization of OCT Family Genes on Tomato Chromosomes

According to the annotation file of the tomato genome, details of all tomato chromosomes were obtained. The relative position of *SlOCT* genes on chromosomes was visualized using TBtools software.

### 2.4. Gene Structure, Protein Conserved Motifs and Domains

Based on the annotation file of the tomato genome, the information on whether the *SlOCT* genes contain introns, the length and number of introns were obtained. For conserved motifs, the MEME database was selected for identification (accessed on 11 February 2025), protein sequences were submitted to the database and the number of motifs was set to the default. Conservative structure domain identification of choice NCBI databases (accessed on 11 February 2025), TBtools software was used to visualize the results together with conserved motifs and gene structures.

### 2.5. Phylogenetic Tree Construction of OCT Family Proteins in Tomato and Arabidopsis

All currently known sequences of OCT proteins in Arabidopsis were obtained from the TAIR database [4,10,11]. The sequences of the above proteins were input into the phylogenetic tree construction plug-in of TBtools software, and the Clustal W method was selected to align the sequences (clustalw, version 2.0.10, software parameters were set to default). The maximum likelihood method was selected for tree construction, and the boot-strap value was set to 10,000. After the program, the data of the phylogenetic tree were submitted to the ITOL website for graphical drawing (accessed on 5 March 2025).

### 2.6. Collinearity Between OCT Family Genes

Genomic data for Arabidopsis (TAIR10), rice (*Oryza sativa* v7.0) and potato (*Solanum tuberosum* v3.0) were obtained from the TAIR and Phytozome databases. The genome assembly and annotation files were analyzed using TBtools software to investigate the collinearity of *SlOCT* genes. The genomes and corresponding annotation files of different plants were submitted to the One Step MCScanX-Super Fast plugin of TBtools software to analyze the synteny between the *OCT* genes of different plants (tomato vs. Arabidopsis/rice/potato).

### 2.7. Identification and Classification of cis-Acting Elements in Promoter

The promoter regions (2000 bp upstream) of *SlOCTs* were screened for *cis*-acting elements using the PlantCARE database (accessed 4 March 2025). The resulting data were graphed using the heatmap plug-in in the TBtools software.

### 2.8. Expression of SlOCT Family Genes Under Cold and Salt Stresses Based on Publicly Available Transcriptome Data

The transcriptome data used in this study were all open-access data obtained from the NCBI GEO database (https://www.ncbi.nlm.nih.gov/gds/, accessed on 4 March 2025). The data download numbers are GSE148887 (cold stress; cultivated tomato Micro-TOM) and PRJNA888477 (salt stress; cultivated tomato M82). FPKM values of *SlOCT* genes were extracted, and TBtools software was used to draw visual heat maps.

### 2.9. qRT-PCR Experiments

Total RNA was extracted from tomato tissues using the Trizol method. Tomato tissues at 0 h, 0.5 h, 2 h, 4 h, 6 h, 12 h, 24 h and 48 h of stresses as described in 2.1 were used in qRT-PCR experiments to define the expression of *SlOCT* family genes under cold and salt stresses; tomato roots 21 days after transfection with the *SlOCT20*-silencing vector (the recombinant vector pTRV2-SlOCT20 mentioned in 2.11) were used to detect *SlOCT20* silencing efficiency. To analyze the tissue-specific expression of the *SlOCT20* gene, samples of roots, stems, leaves, flowers and fruits were collected from 90-day-old tomato seedlings. cDNA was synthesized from total RNA using the HiScript III RT SuperMix for qPCR (+gDNA wiper) kit (Vazyme Biotech Co., Nanjing, China). All qRT-PCR primers were designed using the NCBI website online tool, and all primers used in this study are listed in Table S1. The qRT-PCR experiments were performed using the ChamQ Universal SYBR qPCR Master Mix kit (Vazyme Co., Nanjing, China); the reaction temperature and time referred to the instructions of the kit. Three replicates were performed for each sample. After the qRT-PCR reaction, the specificity of the primers was verified based on the presence or absence of a single, sharp peak in the melting curve. *β-actin* in tomato was chosen as the reference gene [12,13], and the relative expression was calculated using the 2^−∆∆Ct^ method [14].

### 2.10. Analysis of Subcellular Localization

PCR primers were designed according to the CDS sequence (does not contain a stop codon) of the *SlOCT20* gene and the sequence of the pCAMBIA2300-EGFP vector [15]. The restriction sites were *KPN* I and *BamH* I, and the EGFP protein was fused to the C-terminus of SlOCT20; primers were listed in Table S1. The CDS of *SlOCT20* with the addition of a homology arm was amplified using tomato cDNA as a template and ligated to the linearized pCAMBIA2300-EGFP vector using the homologous recombinase product from Vazyme Biotech Co., Ltd. (Nanjing, China). The recombinant vector was transferred into *Agrobacterium tumefaciens* after Sanger sequencing verification by Sangon BioEngineering Co., Ltd. (Shanghai, China). *Agrobacterium tumefaciens* carrying the vector was injected into *Nicotiana benthamiana*, and fluorescence signals in transiently transformed *Nicotiana benthamiana* leaf cells were observed using laser confocal microscopy (1–1000×, Nikon Co., Tokyo, Japan). EGFP was excited using a 488 nm luminescence wavelength.

### 2.11. Silencing of the Tomato SlOCT20 Gene

VIGS targets and primers were designed according to the sequences of the *SlOCT20* gene and vector (Table S1), and PCR was performed. The VIGS target was located in the CDS region of the *SlOCT20* gene (1–300 bp), with a total length of 300 bp. The uniqueness of the target was verified by the BLAST target sequence in the whole tomato genome sequence. PCR amplification system: cDNA from Ailsa Craig 1 μL, upstream and downstream primers 1 μL each, KOD One^TM^ PCR Master Mix (TOYOBO Co., Ltd., Osaka, Japan) 25 μL, ddH_2_O 22 μL. Thirty cycles of 98 °C for 10 s, 56 °C for 5 s and 68 °C for 10 s were performed. The products were subjected to 1% agarose gel electrophoresis to purify the correct bands. The target sequences were ligated into the pNC-pTRV2 empty vector using the Nimble Cloning commercial kit [16]. pTRV2-SlOCT20, empty vector pTRV2-00 and pTRV1, which were verified by Sanger sequencing, were transformed into *Agrobacterium tumefaciens.* Transient transformation was performed using the same method as Chen et al.; bacteria were resuspended using MgCl_2_-IM liquid medium, OD600 was adjusted to 0.6 before injection into tomato seedlings and the seedling age of the tomatoes used was 3 weeks [17]. After 21 days of transformation, the efficiency of *SlOCT20* silencing was identified by qRT-PCR experiments.

### 2.12. Analysis of Physiology

H_2_O_2_ content was measured using the commercial kit H_2_O_2_/2/Y (Keming Co., Suzhou, China). The detection was carried out according to the instructions. The rate of O_2_^−.^ production was detected according to the method proposed by Elstner and Heupel [18]. The PSII maximum photochemical quantum yield (Fv/Fm) was detected using a Plant fluorescence imager (WALZ company, Effeltrich, Germany). The intact tomato leaves were removed and placed on the test table, and the Fv/Fm value was directly detected using the built-in software of the Plant fluorescence imager after switching on.

SOD, POD, CAT activities and MDA content in tomato were detected by kits (SOD/1/Y, POD/1/Y, CAT/1/Y and MDA/1/Y; Keming Co., Suzhou, China). Cadaverine content was detected by Plant Cadaverine Elisa Kit (BLL105866E; Shanghai Baililai Biotechnology Co., Shanghai, China). Three biological replicates were performed for each sample. Individual tomato plants were sampled for each biological replicate. Three parallel technical replicates were performed for each sample.

### 2.13. Statistical Analyses

The experimental data generated in this study were tested for significance; the data of a single independent variable were tested for significance by one-way ANOVA, and the data of two independent variables were tested for significance by two-way ANOVA using IBM SPSS software (Version 22). The means were separated using Fisher’s protected LSD test at the 5% level of probability in two-way ANOVA, and the *t*-test was used in a one-way ANOVA.

## 3. Results

### 3.1. Identification of OCT Family Genes in Tomato

The tomato genome and its annotation files were retrieved from the Phytozome database, while Arabidopsis OCT protein sequences were obtained from the TAIR database. The coding sequences (CDS) of tomato were extracted from the genome file and converted into corresponding protein sequences. These sequences were then subjected to a BLAST search against the Arabidopsis OCT protein sequences. Additionally, HMM files for the OCT proteins were sourced from the Pfam database, and these files were used for HMM-based identification within the tomato protein sequences. By integrating the results from both the BLAST and HMM analyses, a total of 52 *OCT* genes were successfully identified in tomato (Table S2).

### 3.2. Chromosomal Localization of Tomato OCT Genes and Characters of Encoded Protein

**Figure 1:**
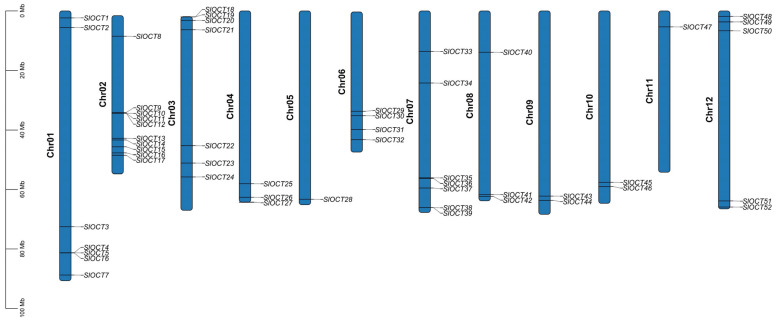
Chromosomal distribution of tomato *OCT* genes. The left scale represents the length of the chromosome.

The tomato *OCT* genes were assigned names from *SlOCT1* to *SlOCT52* based on their positions on the chromosomes, with their chromosomal distribution visualized using TBtools software (Figure 1). Analysis revealed that the *SlOCT* genes are dispersed across all tomato chromosomes, with chromosome 2 containing the most *SlOCT* genes (ten genes) and chromosomes 5 and 11 containing the fewest *SlOCT* genes (only one gene). Most of the *SlOCT* genes were distributed at both ends of the chromosome. Regarding the encoded proteins, the amino acid lengths varied from 251 (SlOCT16) to 738 (SlOCT27), molecular weights ranged from 27.60 kDa (SlOCT16) to 79.33 kDa (SlOCT27), and the isoelectric points varied between 4.60 (SlOCT45) and 10.08 (SlOCT19) (Table S2).

### 3.3. Structure of Tomato OCT Family Genes and Conserved Motifs and Domains of Their Encoded Proteins

**Figure 2:**
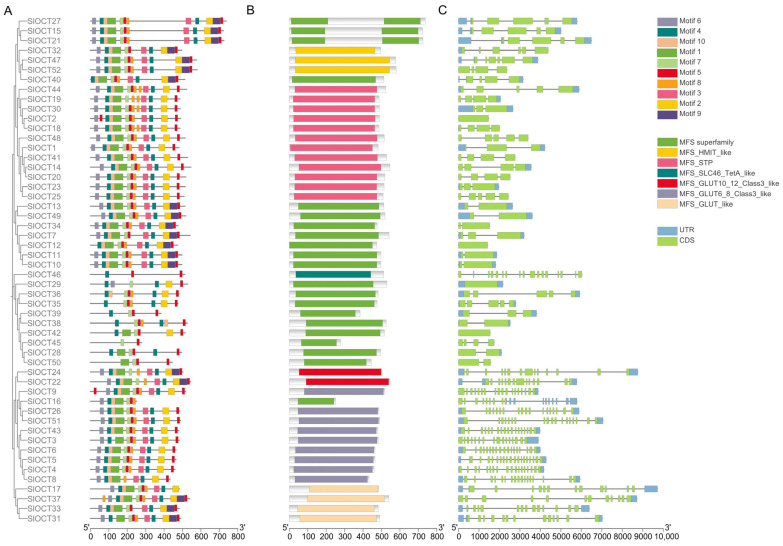
(**A**) Conserved motifs of tomato OCT proteins. (**B**) Conserved domains of tomato OCT proteins. (**C**) Structure of tomato *OCT* genes. The scale lines indicate the lengths of proteins and genes.

The tomato OCT family protein sequences were submitted to the MEME and NCBI databases to identify conserved motifs and domains, respectively. These results, along with the tomato genome annotation file, were processed using TBtools software for structural analysis and visualization of the tomato *OCT* genes. A total of ten conserved motifs (Figure 2A) and seven conserved domains were identified in the tomato OCT family members. A total of 21 SlOCT proteins contained the MFS superfamily domain, 12 SlOCT proteins contained the MFS_-_STP_-_like domain, 9 SlOCT proteins contained the MFS_-_GLUT6_-_8_-_Class3_-_like domain, 4 SlOCT proteins contained the MFS_-_GLUT_-_like domain, 2 SlOCT proteins contained the MFS_-_GLUT10_-_12_-_Class3_-_like domain, 3 SlOCT proteins contained the MFS_-_HMIT_-_like domain and only 1 SlOCT protein contained the MFS_-_SLC46_-_TetA_-_like domain (Figure 2B). In addition, all *SlOCT* genes contained introns (Figure 2C).

### 3.4. Phylogenetic Analysis of OCT Family Proteins

**Figure 3:**
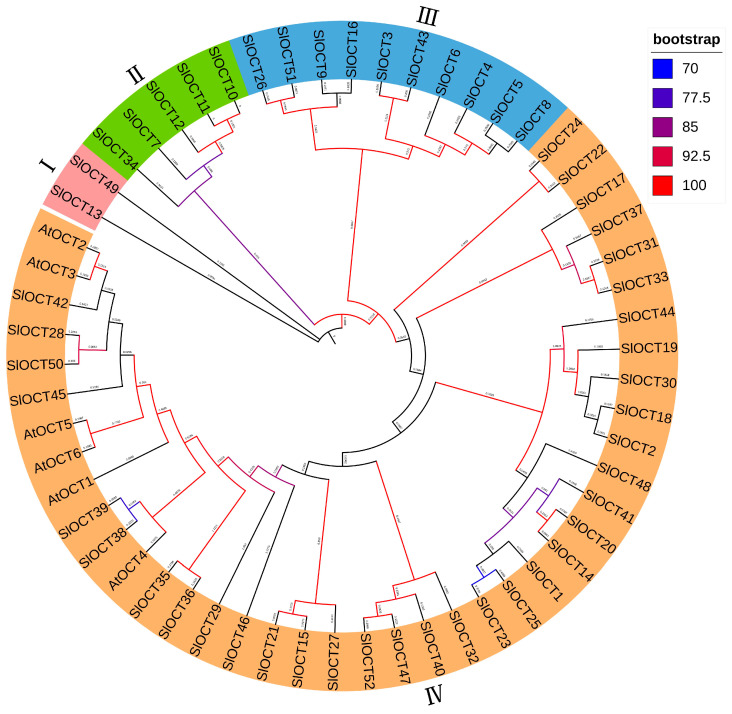
Phylogenetic tree of Arabidopsis and tomato OCT family proteins. The proteins are classified into four groups (I–IV) in the phylogenetic tree.

To reconstruct the phylogenetic relationship, OCT protein sequences from both Arabidopsis (AtOCT1-AtOCT6, TAIR10) and tomato were aligned and used for constructing the phylogenetic tree. The proteins in the phylogenetic tree were classified into four distinct branches. Group I contains two tomato OCT proteins, and the homology of these two proteins is low. Group II contains 5 tomato OCT proteins, Group III contains 10 tomato OCT proteins and Group IV contains 35 tomato OCT proteins and six Arabidopsis OCT proteins (Figure 3), suggesting that some Arabidopsis and tomato OCT proteins may have come from the same ancestor.

### 3.5. Collinearity Analysis of Tomato OCT Family Genes

**Figure 4:**
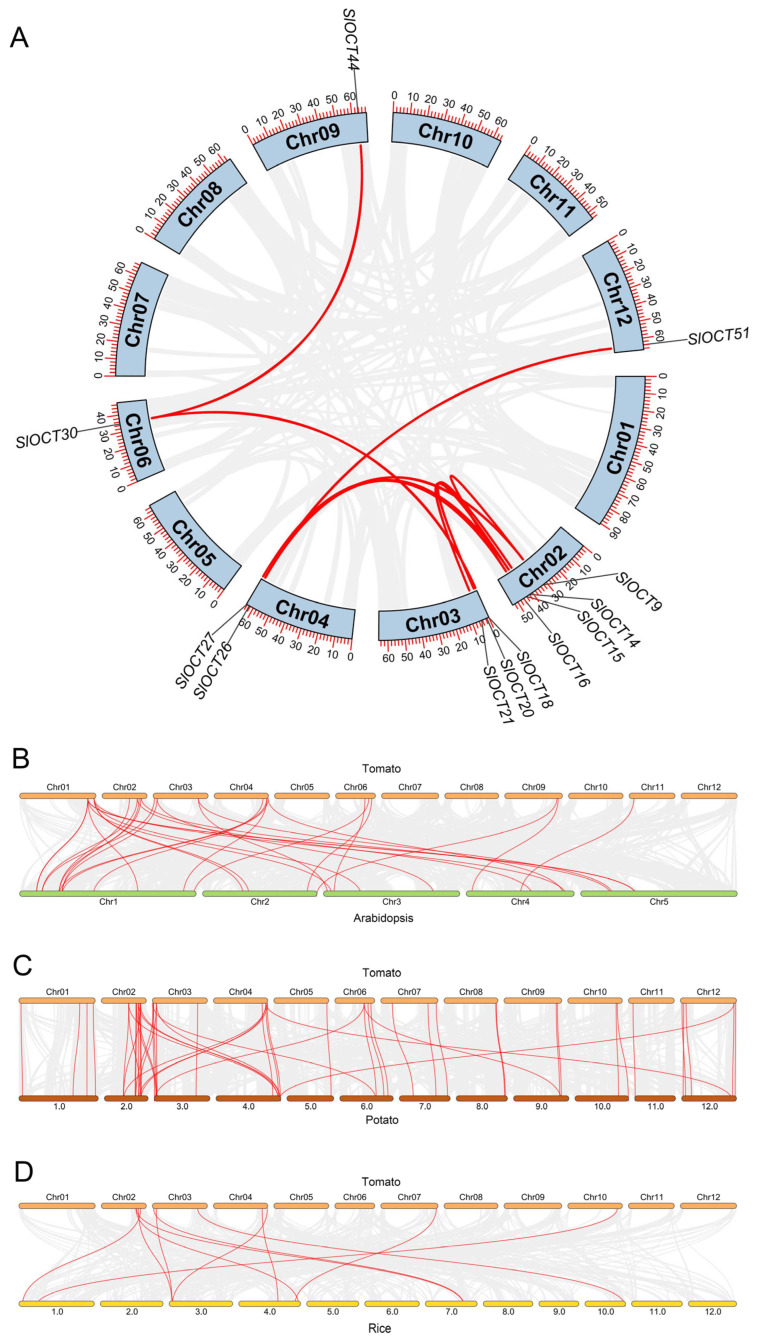
(**A**) Collinearity among the *OCT* family genes in tomato. (**B**) Genome collinearity between tomato and Arabidopsis. (**C**) Genome collinearity between tomato and potato. (**D**) Genome collinearity between tomato and rice. The red lines highlight syntenic *OCT* gene pairs, while the gray lines indicate background genomic collinearity.

By analyzing the genome-wide collinearity of the 52 identified *SlOCT* genes, duplication events within the *OCT* family were investigated. The results revealed nine pairs of collinear tomato *OCT* genes, such as *SlOCT30* and *SlOCT44* (Figure 4A), suggesting that these genes underwent repeated duplication events during evolution. Additionally, synteny analysis was performed to explore the evolutionary relationships between tomato *OCT* genes and those from other plants. The analysis identified 32 syntenic relationships between tomato *OCT* genes and Arabidopsis *OCT* genes (Figure 4B), 54 syntenic relationships with potato OCT genes (Figure 4C), and 11 syntenic relationships with rice *OCT* genes (Figure 4D). Notably, the highest number of syntenic relationships was observed between tomato and potato *OCT* genes, likely due to the close evolutionary relationship between these two solanaceous species.

### 3.6. Analysis of cis-Acting Elements in the Promoters of Tomato OCT Family Genes

**Figure 5:**
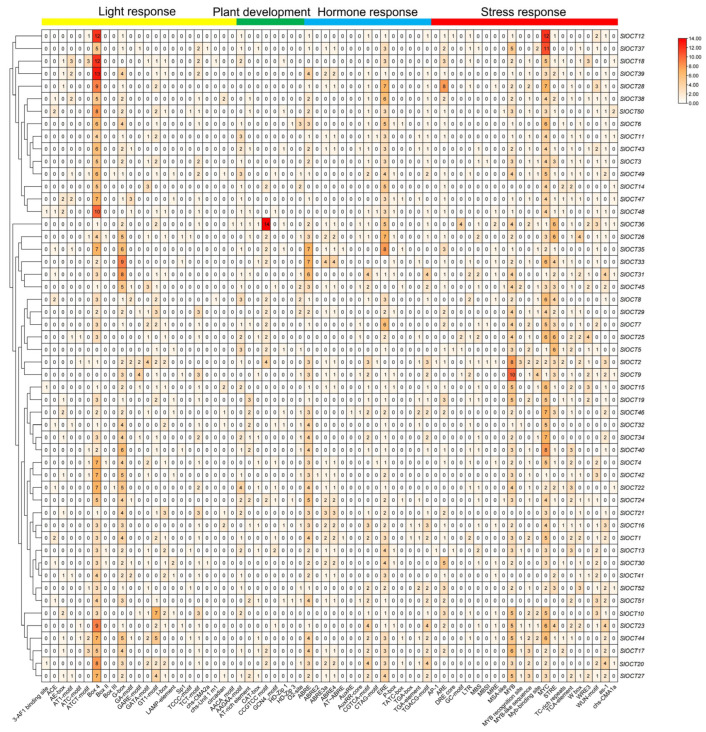
The *cis*-acting elements in promoters of tomato *OCT* family genes. Visual heat maps were drawn using TBtools software. The darker red color of the heat map indicates a greater number of *cis*-acting elements.

To investigate the types, quantities and functions of *cis*-acting elements in the promoter regions of tomato *OCT* family genes, the sequences of the promoter regions of all *OCT* genes were extracted for identification. The identification results were statistically analyzed, and a visual heatmap was generated using TBtools software. In the promoter regions of tomato *OCT* genes, 68 *cis*-acting elements were identified and predominantly assigned to four functional groups: light response, plant development, hormone response, and stress response. Light response and plant development elements, such as Box 4 and CCGTCC motifs, were commonly found in plant gene promoters and were also prevalent in the promoters of tomato *OCT* genes. Several hormone response elements, including ABRE and AuxRE, were detected, suggesting that tomato *OCT* genes may be regulated by certain hormones. Importantly, the promoters of tomato *OCT* genes were particularly rich in *cis*-acting elements associated with stress response, such as MYB and MYC transcription factor binding sites and stress response motifs like the WUN-motif (Figure 5).

### 3.7. Transcriptome Data and qRT-PCR Analysis of Tomato OCT Family Genes Under Salt and Cold Stress

**Figure 6:**
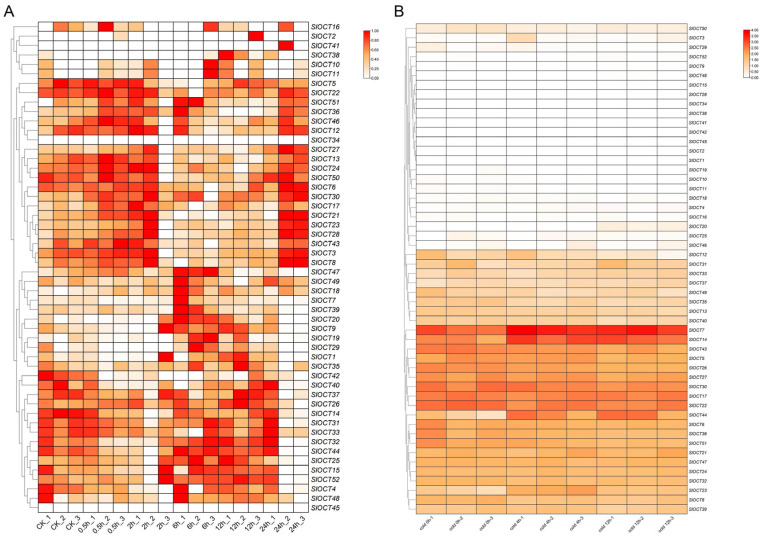
Transcriptomic expression levels of *OCT* Genes under salt (**A**) and cold (**B**) stress. Higher gene expression levels are denoted by darker red colors on the heatmap.

To gain an initial understanding of the expression patterns of *OCT* family genes in tomato under various stresses, we retrieved publicly available transcriptome data from the NCBI database, focusing on representative environmental stresses (salt and cold stress). The FPKM values for the *OCT* family genes were extracted, and a heatmap was generated using TBtools software. Except for *SlOCT34* and *SlOCT45*, all other tomato *OCT* family genes had detected FPKM values in the tomato transcriptome data under salt stress (Figure 6A). Most of the tomato *OCT* family genes showed changes in expression under salt stress, indicating that *OCT* family genes could respond to salt stress. Under cold stress, FPKM values of *SlOCT1/2/15/28/34/42/45* were not detected in transcriptome data, and some of the other *SlOCT* family genes whose FPKM values were detected also showed changes in gene expression (Figure 6B), implying functional diversity of *SlOCT* family genes.

**Figure 7:**
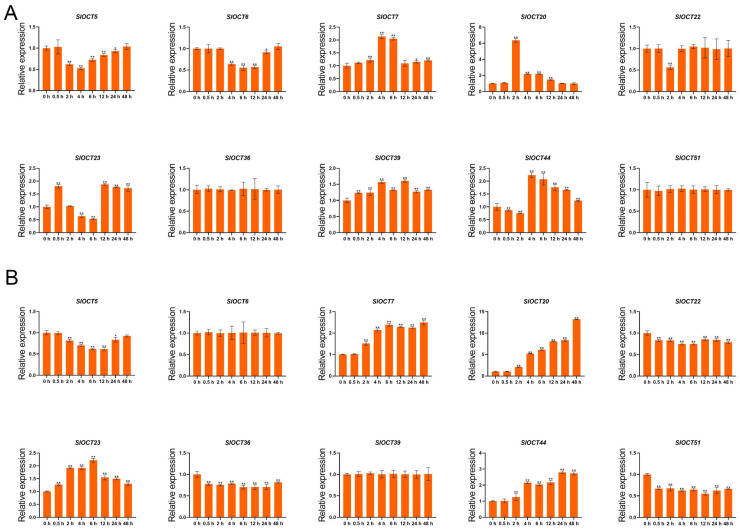
(**A**) Relative expression of tomato *OCT* genes under salt stress. (**B**) Relative expression of tomato *OCT* genes under cold stress. The standard errors in the figures were calculated in triplicate from three samples. One-way analysis of variance was used to calculate the significance between different samples. * means significant, ** means extremely significant.

To further understand the expression patterns of tomato *OCT* family genes under different stresses and to explore the potential key genes that function under salt or cold stress, we selected genes with stable FPKM values and relatively regular expression in the salt and cold stress transcriptome data for stress qRT-PCR analysis (*SlOCT5/6/7/20/22/23/36/39/44/51*). The results showed that salt stress up-regulated the expression of *SlOCT7*, *SlOCT20* and *SlOCT39* and down-regulated the expression of *SlOCT5*, *SlOCT6* and *SlOCT22*. The expression of *SlOCT23* was first up-regulated and then down-regulated, and the expression of *SlOCT44* was first down-regulated and then up-regulated. There was no significant change in the expression of *SlOCT36* and *SlOCT51* (Figure 7A). Under cold stress, *SlOCT7*, *SlOCT20*, *SlOCT23* and *SlOCT44* were up-regulated, *SlOCT5*, *SlOCT22*, *SlOCT36* and *SlOCT51* were down-regulated, and *SlOCT6* and *SlOCT39* expressions did not change significantly (Figure 7B). Notably, the expression of *SlOCT20* was significantly up-regulated under both salt and cold stress, and the up-regulation was the highest. These findings led to the hypothesis that *SlOCT20* is involved in salt and cold stress responses in tomato, leading to its selection as a prime candidate for functional validation.

### 3.8. Analysis of SlOCT20 Protein Subcellular Localization

**Figure 8:**
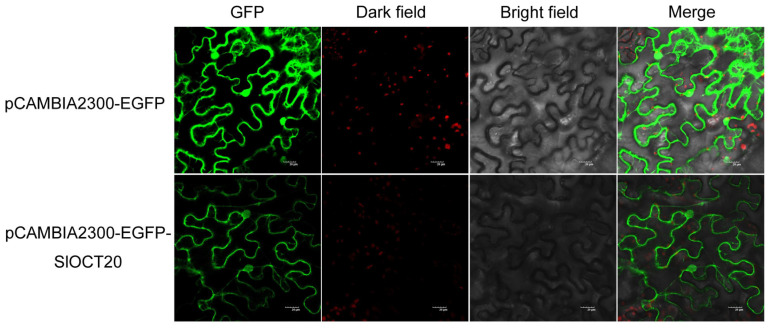
Subcellular localization of tomato SlOCT20 protein. The green signal is GFP fluorescence under laser confocal microscopy, and the red signal is chloroplast autofluorescence under dark field.

The *SlOCT20* was ligated to the vector pCAMBIA2300-EGFP, and the EGFP tag was fused to the C-terminus of the SlOCT20 protein. The validated vector pCAMBIA2300-EGFP-SlOCT20 and the empty vector pCAMBIA2300-EGFP were transiently transformed into epidermal cells of *Nicotiana benthamiana* leaves, respectively. Laser confocal microscopy was used to visualize fluorescence. The green fluorescence signal of EGFP and chloroplast autofluorescence in the dark field could be clearly observed in the transformed empty vector group, indicating that the transient transformation was successful. The green fluorescence signal of EGFP in the transformed recombinant vector pCAMBIA2300-EGFP-SlOCT20 group mainly appeared in the cell membrane, indicating that the SlOCT20 protein was mainly localized in the cell membrane (Figure 8).

### 3.9. Silencing of SlOCT20 Reduced the Cold Tolerance of Tomato

VIGS was used to silence *SlOCT20* in tomato to understand the function of tomato *SlOCT20* under salt and cold stresses. The silencing efficiency of *SlOCT20* was identified 21 days after transient transformation. The expression of *SlOCT20* in most (8/10) *SlOCT20*-silenced tomatoes was significantly lower than that in the control (Figure S1), indicating that *SlOCT20* has been silenced in tomatoes. Tomatoes with higher silencing efficiency were chosen for subsequent experiments (pTRV2-*SlOCT20*#4, 7, 8, 9).

**Figure 9:**
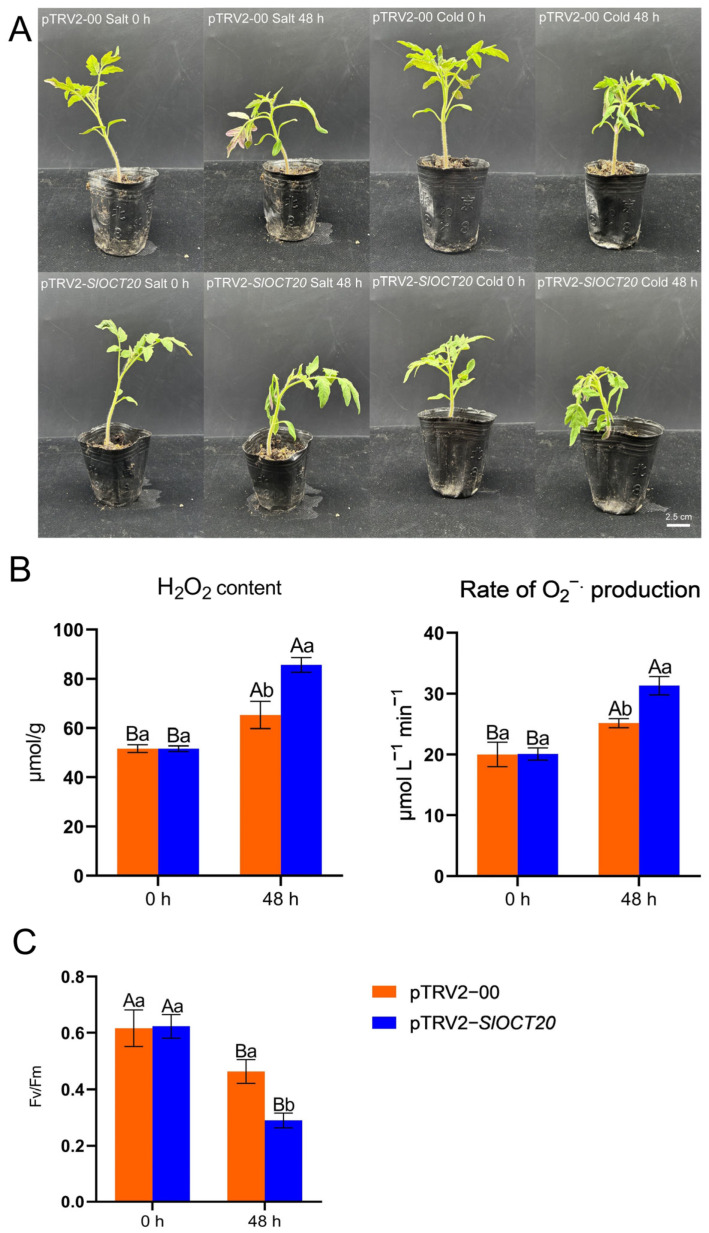
(**A**) Phenotypic observation of *SlOCT20*-silenced tomato under salt and cold stress. (**B**) H_2_O_2_ content and rate of O_2_^−.^ production of *SlOCT20*-silenced tomato under cold stress. (**C**) Fv/Fm value of *SlOCT20*-silenced tomato under cold stress. The standard errors in the figures were calculated in triplicate from three samples. Two-factor analysis of variance was used to calculate the significance between different samples. Capital marks represent significance between samples at different time points, and lowercase marks represent significance between the control and *SlOCT20*-silenced tomatoes.

Phenotypic differences between control and *SlOCT20*-silenced tomatoes were analyzed after 48 h of salt and cold stresses. After 48 h of salt and cold stresses, both control and *SlOCT20*-silenced tomatoes showed a wilting phenotype, but there was no significant difference in phenotype between the control and *SlOCT20*-silenced tomatoes after salt stress. However, compared with the control, *SlOCT20*-silenced tomatoes exhibited a less cold-tolerant phenotype after cold stress (Figure 9A). Reactive oxygen species accumulation (H_2_O_2_ and O_2_^−.^) and PSII maximum photochemical quantum yield (Fv/Fm) are often used to evaluate the degree of plant damage under cold stress. H_2_O_2_ content, rate of O_2_^−.^ production, and Fv/Fm were measured in control and *SlOCT20*-silenced tomato leaves after cold stress. H_2_O_2_ content and rate of O_2_^−.^ production in *SlOCT20*-silenced tomatoes were higher than that in the control (Figure 9B), and the Fv/Fm value was lower than that in the control (Figure 9C). This suggests that *SlOCT20*-silenced tomatoes were more severely damaged and less tolerant to cold after cold stress.

### 3.10. Tomato SlOCT20 May Positively Regulate Cold Tolerance by Regulating Cadaverine Transport from Soil to Roots

**Figure 10:**
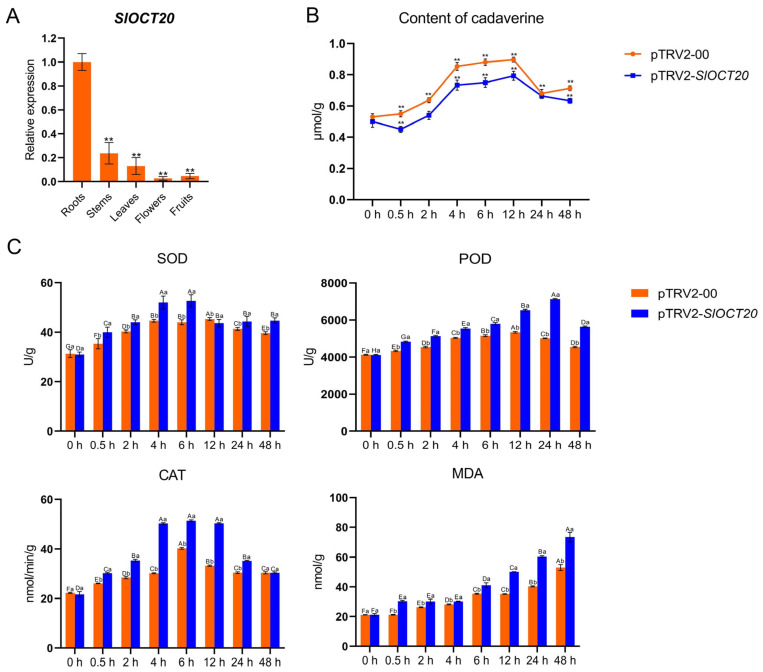
(**A**) Differential expression of the *SlOCT20* gene in different organs of tomato. (**B**) Cadaverine content in *SlOCT20*-silenced tomato roots under cold stress. The standard errors in the figures were calculated in triplicate from three samples. One-way analysis of variance was used to calculate the significance between different samples. ** means extremely significant. (**C**) SOD, POD, CAT activities and MDA content in the roots of *SlOCT20*-silenced tomatoes under cold stress. The standard errors in the figures were calculated in triplicate from three samples. Two-factor analysis of variance was used to calculate the significance between different samples. Capital marks represent significance between samples at different time points, and lowercase marks represent significance between the control and *SlOCT20*-silenced tomatoes.

As mentioned above, OCT family proteins can transport some organic cations, such as carnitine and cadaverine, and cadaverine has been reported to enhance cold tolerance by mitigating oxidative damage in plants. We examined the expression of the *SlOCT20* gene in different tomato organs, and although the *SlOCT20* gene is expressed in different tomato organs, the highest expression level is found in the root system (Figure 10A). After cold stress, the expression of *SlOCT20* was upregulated in tomato roots, and the silencing of *SlOCT20* had a negative impact on this response pattern (Figure S2). The results of cadaverine detection in the roots of control and *SlOCT20*-silenced tomatoes showed that cadaverine content in the roots of *SlOCT20*-silenced tomatoes was significantly lower than that of control tomatoes under cold stress (Figure 10B). SOD, POD, CAT activities and MDA content in the roots of control and *SlOCT20*-silenced tomatoes were increased under cold stress. In addition, SOD, POD, CAT activities and MDA content in *SlOCT20*-silenced tomatoes were significantly higher than those in control tomatoes (Figure 10C), indicating that the oxidative damage of *SlOCT20*-silenced tomatoes was more serious under cold stress. In conclusion, tomato *SlOCT20* may enhance cold tolerance by regulating cadaverine accumulation in roots and reducing oxidative damage.

## 4. Discussion

Tomatoes often suffer from various environmental stresses during their growth and development, such as cold stress and saline-alkali stress [19]. Tomatoes prefer warmth, and cold stress is extremely harmful to their growth and development. The regulation mechanism of plants to cold stress is mainly through Ca^2+^, hormones and other signaling pathways to activate the expression of cold resistance genes, such as *CBFs* and *ICEs*. In addition, other indirect mechanisms also regulate plant tolerance to cold stress [20]. The transmembrane transport of cations not only plays a role in mineral absorption, osmotic pressure regulation, cell expansion, and signal transduction, but also relates to plant stress tolerance [3]. In this study, all of the *OCT* family genes were identified in the tomato genome version 4.0 (Table S2), and the structures of these *OCT* genes and encoded proteins were characterized (Figure 2). Most of the *SlOCT* genes are unevenly distributed at both ends of the chromosome (Figure 1). This phenomenon is frequently seen in other similar studies [21,22,23,24,25] and may be related to gene evolutionary expansion. Phylogenetic and collinearity analysis of OCT proteins and *OCT* family genes in tomato and different plants showed that *OCT* genes in tomato and *OCT* genes in other plants (such as Arabidopsis, rice and potato) may come from the same ancestor, and *SlOCT* family genes were repeatedly generated during evolution (Figure 3 and Figure 4). In addition, the identification of *cis*-acting elements in the promoter of tomato *OCT* family genes also implies a correlation between gene function and environmental stress in this family.

Under stress, plants will adjust their gene expression to adapt to stress and reduce stress damage. We found that many tomato *OCT* family genes could respond to salt and cold stress using publicly available omics data and qRT-PCR validation under salt and cold stresses (Figure 6 and Figure 7). We selected the *SlOCT20* gene, whose expression was significantly up-regulated and with the highest up-regulation fold under salt stress and cold stress, as the hypothetical key gene for later functional studies. We cloned the full-length CDS of *SlOCT20* in tomato and found that the encoded protein was mainly localized to the cell membrane (Figure 8). The localization of tomato OCT protein obtained in this study is consistent with the localization of Arabidopsis OCT protein [5]. The expression of SlOCT20 protein on the plasma membrane suggests that it may have an ion transport function. We treated the constructed *SlOCT20*-silenced tomatoes with salt and cold stress, and the phenotype of tomato plants treated with stress for 48 h showed that under cold stress, *SlOCT20*-silenced tomatoes exhibited a phenotype that was less cold-tolerant compared to the control tomatoes (Figure 9A). We also tried to demonstrate the reduction of cold tolerance in *SlOCT20*-silencing tomatoes from other physiological indicators. It has been shown that plants will produce a strong burst of reactive oxygen species in cells after stress [26], and excessive bursts of reactive oxygen species in a short period of time can impair cell function [27]. Therefore, tissue-reactive oxygen species content is often used to assess the severity of tissue damage caused by stress. The results of H_2_O_2_ content and the rate of O_2_^−.^ production showed that the above indicators in *SlOCT20*-silenced tomatoes were higher than those in the control (Figure 9B), indicating that the oxidative stress of *SlOCT20*-silenced tomatoes was more serious after cold stress. In addition, the Fv/Fm can reflect photosynthetic capacity and is often used to assess plant health under cold stress. Lower values of Fv/Fm indicate weaker photosynthetic capacity and poorer health of the plant [28]. The Fv/Fm of *SlOCT20*-silenced tomato plants under cold stress was significantly lower than that of the control plants (Figure 9C). The PSⅡ maximum photochemical quantum yield also showed that the silencing of the *SlOCT20* gene reduced the cold tolerance of tomato plants.

We tried to understand the underlying mechanism of *SlOCT20* in regulating tomato cold tolerance. By detecting the expression levels of *SlOCT20* in different organs of tomato, we found that *SlOCT20* was most highly expressed in tomato roots (Figure 10A). After cold stress, the expression of *SlOCT20* was upregulated in tomato roots, and the silencing of *SlOCT20* had a negative impact on this response pattern (Figure S2). Combined with the results of localization of SlOCT20 protein on the cell membrane, we hypothesized that the tomato SlOCT20 protein may play a role in the uptake of some beneficial organic cations in soil by the root system. Cadaverine, as an organic cation widely present in soil, plays an active role in plant stress resistance and antioxidant processes [7,8]. Our results showed that cadaverine content in the roots of *SlOCT20*-silenced tomato was significantly lower than that of the control under cold treatment (Figure 10B). In addition, silencing of *SlOCT20* significantly increased SOD, POD, CAT activities and MDA content in tomato roots under cold stress (Figure 10C). The complete metabolic pathway formed by the above-mentioned antioxidant enzymes in plants can convert reactive oxygen species into biologically harmless substances. Their activities increase rapidly in the early stage of stress to remove harmful substances, and the more severe the oxidative damage to the cells, the more intense the burst of enzyme activity [29,30,31]. Previous studies have shown that cadaverine absorption, transport, and accumulation in plants are positively correlated with antioxidant systems under stress [32]; therefore, we speculate that silencing *SlOCT20* may lead to a reduction in cadaverine accumulation in roots, thereby reducing the antioxidant capacity of tomato under cold stress.

## 5. Conclusions

In this study, 52 *OCT* family genes were identified in tomato for the first time, and a detailed bioinformatics analysis of these genes was conducted. A key gene regulating cold stress tolerance, *SlOCT20*, was identified. SlOCT20 was mainly localized in the cell membrane and positively regulates cold tolerance in tomato.

## Data Availability

The raw data supporting the conclusions of this article will be made available by the authors on request.

## Supplementary Materials

The following supporting information can be downloaded at: https://www.mdpi.com/article/10.3390/biology15020176/s1, Figure S1: Detection of *SlOCT20* silencing efficiency; Figure S2: Expression of *SlOCT20* in tomato rootlets under cold stress; Table S1: All primers in this article; Table S2: Information of *OCT* family members in tomato.

## Abbreviations

The following abbreviations are used in this manuscript:

OCT	Organic cation transporters
HMM	Hidden Markov model
CDS	Coding sequence
