# Supplementary material for: Identification of OCT Family Genes in Tomato (Solanum lycopersicum) and Function of SlOCT20 Under Cold Stress
Source: Biology (Basel). 2026 Jan 18;15(2):176. doi: 10.3390/biology15020176 (PMC12837769; doi:10.3390/biology15020176)
Supplement: Supplementary file 1 [file biology-15-00176-s001.zip › Table S1.pdf]

Table S1 All primers in this article

Name	Sequence
SlOCT1-qF	CGATTGGGAATCGAGGGAGA
SlOCT1-qR	CGATCGGACTTCAAAACCACG
SlOCT2-qF	ATTCCCGCGGTGATTCTGTT
SlOCT2-qR	TTCATGAGCGTGGACTTCCC
SlOCT3-qF	GCTGTAGTGCAGGTTCCGAT
SlOCT3-qR	CCAACGAATGTCCCTGTTGC
SlOCT4-qF	AAGACGGCCTGTTCTGATGG
SlOCT4-qR	GTGGCAGTGAGCTGACTCAT
SlOCT5-qF	CGCTGACTCTTGGCTTCTCA
SlOCT5-qR	TTTCGACAACCACCGAGGAG
SlOCT6-qF	CTGGCGTCTATTGGCTGTGA
SlOCT6-qR	TTTCCACTGGCCAGCCTTAG
SlOCT7-qF	AGTCACCGCGTTGGTTAGTT
SlOCT7-qR	GTCGTTGCAGTTCTCCGGTA
SlOCT8-qF	ATATCCGAGGATCTATTCCTTGC
SlOCT8-qR	AGTCATCGCCTCTTAGACATCG
SlOCT9-qF	GGTGCAATGGTTGGTGCAAT
SlOCT9-qR	AATGGCGAACCATCCGCTTA
SlOCT10-qF	TGATTATGTTGGGCGTCGCT
SlOCT10-qR	ACCAACACCAACTCCAGCAA
SlOCT11-qF	TGATTATGTTGGGCGTCGCT
SlOCT11-qR	ACCAACACCAACTCCAGCAA
SlOCT12-qF	GTGCTGGAAGAACCTCCGAT
SlOCT12-qR	ACACCAACTCCAGCAACACA
SlOCT13-qF	TCTGTGCTTCTTGGATATGATGT
SlOCT13-qR	TCCTCCTGCTAAACTCCCCA
SlOCT14-qF	TGTTCTGTGATCAGCTCGGA
SlOCT14-qR	TGGCAGCAACAATGCAAGTC
SlOCT15-qF	GTACAGAAGGGCCTCCAACC
SlOCT15-qR	GCCAACCACCACCTATACCC
SlOCT16-qF	TGTTGGTGCAATGATTGGCG
SlOCT16-qR	CAGTGGCAAATGAAACGGCA
SlOCT17-qF	ACAGTTCTTGCTGGTGCCTT
SlOCT17-qR	ACGCACCAACTGAAAGTGGA
SlOCT18-qF	TCTCCGCTTGGATCGTTGTC
SlOCT18-qR	GGTGCTGCATCCAGACTCTT
SlOCT19-qF	GTGGTGTTTCTCGTGTGTGC
SlOCT19-qR	GAGCATGTTTGTGCTGACCG
SlOCT20-qF	CCTCCTCCCAGAGACTCCAA
SlOCT20-qR	CTCGAGGCTTCACTAGCCAC
SlOCT21-qF	GAGAGCGTCTTGGGTCAACA
SlOCT21-qR	CATGCTTGACTCCTGGCTCA

SlOCT22-qF	GTGGTCAGCGGTTCCCTTTA
SlOCT22-qR	TGGAGCACAGGCAGTTAGTG
SlOCT23-qF	CAACAGGTGACGGGGATCAA
SlOCT23-qR	CCTACCACACCTGTCACCAC
SlOCT24-qF	TGGTGGCTTCCTTCATCACC
SlOCT24-qR	CACCAATTGCAGCTCCCCTA
SlOCT25-qF	GCTCATTTTGGGTCGCGTTT
SlOCT25-qR	ATGCACCACGCCATTTTGAC
SlOCT26-qF	AGTGACTGCACCGTTGCTTA
SlOCT26-qR	TCTCCGGTACCCAAATTGCC
SlOCT27-qF	GTCAACCTGGGGAGTGTAGC
SlOCT27-qR	GATGCACAAACCACGAACCC
SlOCT28-qF	CATGTCACTAGCCGGAACCA
SlOCT28-qR	GTCCACGCCATTGATTCCCT
SlOCT29-qF	CATGTCACTAGCCGGAACCA
SlOCT29-qR	GTCCACGCCATTGATTCCCT
SlOCT30-qF	CATCGAACATGCACCCCAAC
SlOCT30-qR	TAGTGCAGCTTTGCCCTGTT
SlOCT31-qF	GAGGTGCTCTTTTGGGCTCT
SlOCT31-qR	TTGCGGCACTCATAGAAGCA
SlOCT32-qF	GGTGGCATGTCTGCTACTGT
SlOCT32-qR	GAACACGGCGACAAACACAA
SlOCT33-qF	CTGGTCTCCTCCTCCCAGAA
SlOCT33-qR	AGCGCAAGAACATCAGTCCA
SlOCT34-qF	GATGGGCTTTGCTCCTTCCT
SlOCT34-qR	AACCACGAGTCATAGCAGGC
SlOCT35-qF	AGTGACGGCTGTATGTGCAA
SlOCT35-qR	CCATACACAAGTCCACCCCC
SlOCT36-qF	TGCGGGTCTTGGATCAATGG
SlOCT36-qR	GCTCCAATAAGCATGCCAGC
SlOCT37-qF	CAAGGCTGGGAGACTCGATG
SlOCT37-qR	TCTAGGAGCTCCAGCCAACT
SlOCT38-qF	AAGTATTGCGGGGAGTTCGG
SlOCT38-qR	CGTCACATAATGCCACGCTG
SlOCT39-qF	TCTTCCAAACGTGGCGATCA
SlOCT39-qR	TGGCTTTCTACCAAGTTCGTCA
SlOCT40-qF	CACTTGTTACAGCGGGGCTA
SlOCT40-qR	AATCACAACACCGCACAAGC
SlOCT41-qF	GGGGTTGGCGTTTGAGTCTA
SlOCT41-qR	CGTCCCCTTTACCACGTTCA
SlOCT42-qF	AGGTGACTCGATTGGTCGAAA
SlOCT42-qR	CAGCGCGACCATATCCACTA
SlOCT43-qF	TCCGTGGGTTGTGATGTCAG
SlOCT43-qR	TGAAAGTGTAGGAGCACGCC

SlOCT44-qF	GTCATCACCGGAGCAGTCAA
SlOCT44-qR	ACATTTGGACCCCAGCTTCC
SlOCT45-qF	GCCTCCCATCATCCTCCTTC
SlOCT45-qR	AATGCCTTCCCACGAGCTTT
SlOCT46-qF	GACCCTCAGACCCTTTCTGC
SlOCT46-qR	AATTCCGTCAACACGAGCCT
SlOCT47-qF	CTCACAAAGCTGCGAAGACG
SlOCT47-qR	ACAGCAGCCCTCCTATACCA
SlOCT48-qF	GCTGCCATTGGAGGTTCTCT
SlOCT48-qR	TGTTTCCTTTTGTACACTTCTGGG
SlOCT49-qF	GTGGGGATTGGACCTGTCTG
SlOCT49-qR	CCAGCCCACTGCACATTCTA
SlOCT50-qF	TGTTTGTGTCTGCATCACGC
SlOCT50-qR	ACCACTTCAATCCTGTCATCAA
SlOCT51-qF	GTTGGCCAAGATGGGTCTGA
SlOCT51-qR	TCGGCTTGTGGATGCTACAG
SlOCT52-qF	TGCTGGTGCAATTTTCGGTG
SlOCT52-qR	ATGATCAGCCAAGGAGCTGG
SlOCT20-2300F	GGACGAGCTCGGTACCATGGCTGGTGGTGGTGGAA
SlOCT20-2300R	CGACTCTAGAGGATCCATTTGCTCTCCCCTTTGCCATTT
SlOCT20-TRV2F	agtggtctctgtccagtcctATGGCTGGTGGTGGTGG
SlOCT20-TRV2R	ggtctcagcagaccacaagtTACGACCGATGAACATAGTGCAG
