# Supplementary material for: Identification of OCT Family Genes in Tomato (Solanum lycopersicum) and Function of SlOCT20 Under Cold Stress
Source: Biology (Basel). 2026 Jan 18;15(2):176. doi: 10.3390/biology15020176 (PMC12837769; doi:10.3390/biology15020176)
Supplement: Supplementary file 1 [file biology-15-00176-s001.zip › Table S2.pdf]

Table S2 Information of *OCT* family members in tomato

Gene name	geneID	Chr	start	end	aalen	MolWt	pI
SlOCT1	Solyc01g008240.4.1	Chr01	2316569	2320787	482	52607.48	9.42
SlOCT2	Solyc01g010530.1.1	Chr01	5586241	5587710	489	54132.23	9.09
SlOCT3	Solyc01g080680.3.1	Chr01	72502052	72505955	483	52327.61	6.07
SlOCT4	Solyc01g098490.3.1	Chr01	81249236	81253409	460	49537.4	4.98
SlOCT5	Solyc01g098500.3.1	Chr01	81267057	81271343	465	50078.41	6.28
SlOCT6	Solyc01g098560.3.1	Chr01	81307989	81311984	465	50142.15	6.51
SlOCT7	Solyc01g109460.3.1	Chr01	88750978	88754182	541	59047.37	9.33
SlOCT8	Solyc02g005180.3.1	Chr02	6942392	6948324	433	46766.72	6.72
SlOCT9	Solyc02g062750.4.1	Chr02	32530188	32534080	517	56233.98	9.15
SlOCT10	Solyc02g062860.3.1	Chr02	32823487	32825303	497	54309.14	9.44
SlOCT11	Solyc02g062870.3.1	Chr02	32835760	32837632	497	54308.2	9.64
SlOCT12	Solyc02g062890.3.1	Chr02	32864002	32865426	474	51250.96	9.58
SlOCT13	Solyc02g078600.3.1	Chr02	41238788	41241430	513	55327.05	6.88
SlOCT14	Solyc02g079220.4.1	Chr02	41814355	41817906	546	60588.43	9.36
SlOCT15	Solyc02g082410.4.1	Chr02	44097658	44102663	723	77716.31	5.17
SlOCT16	Solyc02g085170.4.1	Chr02	46166115	46171903	251	27596.39	8.02
SlOCT17	Solyc02g086160.4.1	Chr02	46924664	46934384	484	50760.17	9.94
SlOCT18	Solyc03g005140.2.1	Chr03	101708	103714	487	53815.81	9.5
SlOCT19	Solyc03g005150.3.1	Chr03	105255	107309	487	53585.93	10.08
SlOCT20	Solyc03g006650.2.1	Chr03	1266348	1268873	518	56904.08	8.71
SlOCT21	Solyc03g032040.3.1	Chr03	4452839	4459329	725	78094.92	4.89
SlOCT22	Solyc03g078000.3.1	Chr03	43375004	43380785	545	58399.66	9.18
SlOCT23	Solyc03g093410.3.1	Chr03	49257553	49259519	514	55911.7	9.64
SlOCT24	Solyc03g096950.4.1	Chr03	53902565	53911323	503	53750.09	4.84
SlOCT25	Solyc04g074070.2.1	Chr04	58040687	58043123	509	55554.19	9.69
SlOCT26	Solyc04g080460.3.1	Chr04	62606195	62612086	486	52598.91	9.39
SlOCT27	Solyc04g082700.3.1	Chr04	64257380	64263168	738	79331.41	4.77
SlOCT28	Solyc05g053860.4.1	Chr05	63280226	63282326	496	55353.67	7.87
SlOCT29	Solyc06g051860.3.1	Chr06	33343688	33345860	529	59037.82	8.73
SlOCT30	Solyc06g054270.3.1	Chr06	34878094	34880756	489	53684.58	8.81
SlOCT31	Solyc06g066600.3.1	Chr06	39480021	39487046	491	52648.93	7.47
SlOCT32	Solyc06g073420.3.1	Chr06	42914199	42918579	496	53355.41	5.1
SlOCT33	Solyc07g020790.4.1	Chr07	13558548	13564939	483	52320.93	8.76
SlOCT34	Solyc07g024030.3.1	Chr07	24208846	24210358	477	51742.09	8.24
SlOCT35	Solyc07g042690.4.1	Chr07	56067914	56070711	476	51721.16	7.31
SlOCT36	Solyc07g042900.4.1	Chr07	56271419	56277341	482	53072.64	7
SlOCT37	Solyc07g049310.3.1	Chr07	59479930	59488648	540	58974.45	6.44
SlOCT38	Solyc07g063920.3.1	Chr07	66085251	66087780	526	57216.82	7.81
SlOCT39	Solyc07g063930.4.1	Chr07	66088300	66092113	384	41699.91	7.5
SlOCT40	Solyc08g048290.4.1	Chr08	13834187	13837343	513	55671.93	8.15
SlOCT41	Solyc08g080300.1.1	Chr08	61721710	61724478	528	58149.5	9.41

SlOCT42	Solyc08g081090.1.1	Chr08	62322583	62324133	516	56777.61	7.24
SlOCT43	Solyc09g074230.4.1	Chr09	62217205	62221188	480	51987.98	7.53
SlOCT44	Solyc09g075820.3.1	Chr09	63647212	63653104	523	57575.62	9.1
SlOCT45	Solyc10g074850.3.1	Chr10	57606062	57607808	279	31002.99	4.6
SlOCT46	Solyc10g076940.3.1	Chr10	58983544	58989570	512	56223.6	6.51
SlOCT47	Solyc11g012450.2.1	Chr11	5341232	5345114	577	62961.51	9.62
SlOCT48	Solyc12g008320.2.1	Chr12	1784113	1787515	515	56475.57	8.77
SlOCT49	Solyc12g010690.2.1	Chr12	3665633	3669240	519	55983.69	5.83
SlOCT50	Solyc12g017380.3.1	Chr12	6674296	6675867	445	49665.91	6.26
SlOCT51	Solyc12g089180.2.1	Chr12	63859759	63866818	490	52809.88	8.41
SlOCT52	Solyc12g099070.1.1	Chr12	65881960	65884323	580	63071.79	8.83
