# Supplementary figures and images for: Identification of OCT Family Genes in Tomato (Solanum lycopersicum) and Function of SlOCT20 Under Cold Stress
Source: Biology (Basel). 2026 Jan 18;15(2):176. doi: 10.3390/biology15020176 (PMC12837769; doi:10.3390/biology15020176)

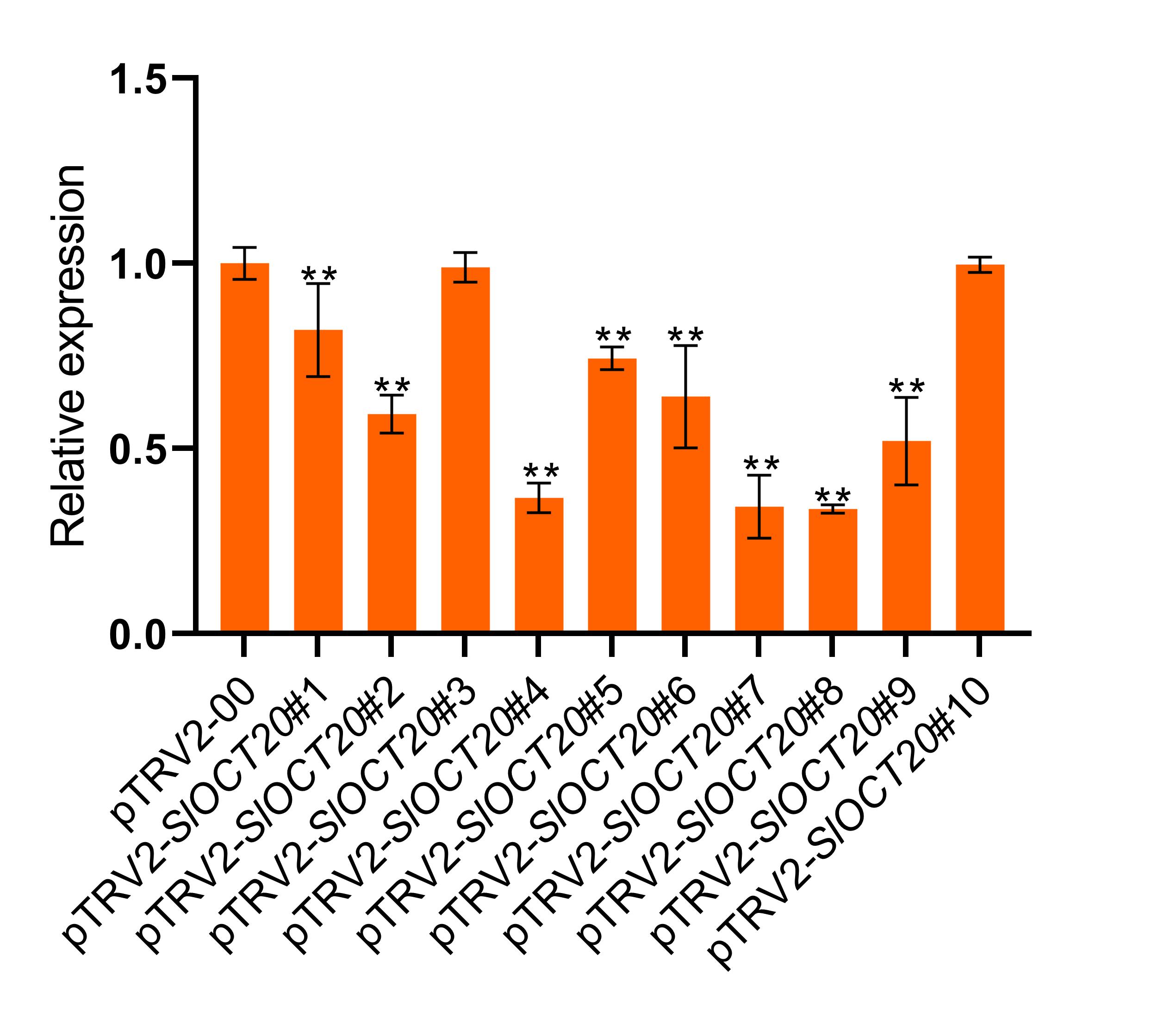

Supplement: Supplementary file 1 [file biology-15-00176-s001.zip › Figure S1.jpg]

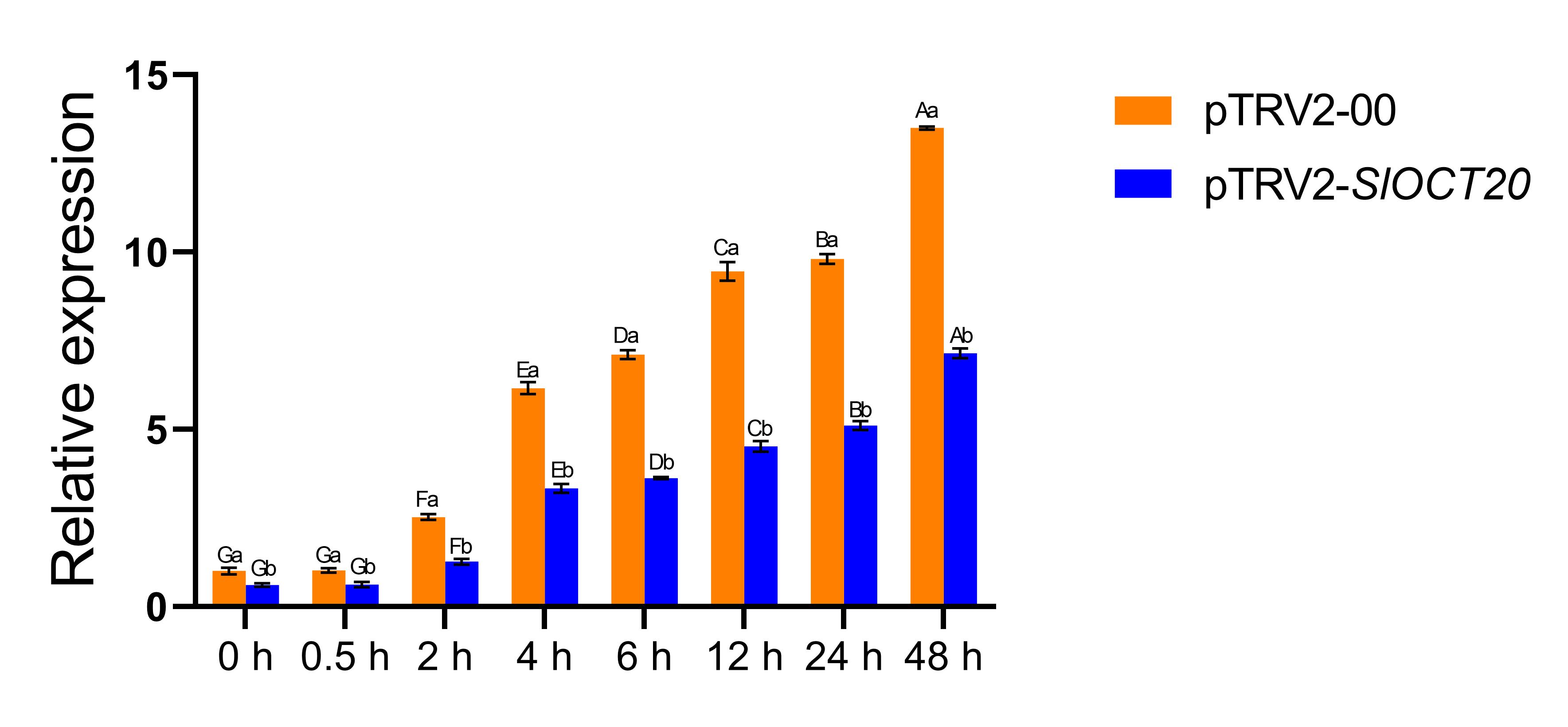

Supplement: Supplementary file 1 [file biology-15-00176-s001.zip › Figure S2.jpg]
